# Enhanced Nasal Mucosal Delivery and Immunogenicity of Anti-Caries DNA Vaccine through Incorporation of Anionic Liposomes in Chitosan/DNA Complexes

**DOI:** 10.1371/journal.pone.0071953

**Published:** 2013-08-20

**Authors:** Liulin Chen, Junming Zhu, Yuhong Li, Jie Lu, Li Gao, Huibi Xu, Mingwen Fan, Xiangliang Yang

**Affiliations:** 1 National Engineering Research Center for Nanomedicine, College of Life Science and Technology, Huazhong University of Science and Technology, Wuhan, PR China; 2 Key Laboratory for Oral Biomedical Engineering of Ministry of Education, School and Hospital of Stomatology, Wuhan University, Wuhan, PR China; Institute for Frontier Medical Sciences, Kyoto University, Japan

## Abstract

The design of optimized nanoparticles offers a promising strategy to enable DNA vaccines to cross various physiological barriers for eliciting a specific and protective mucosal immunity via intranasal administration. Here, we reported a new designed nanoparticle system through incorporating anionic liposomes (AL) into chitosan/DNA (CS/DNA) complexes. With enhanced cellular uptake, the constructed AL/CS/DNA nanoparticles can deliver the anti-caries DNA vaccine pGJA-P/VAX into nasal mucosa. TEM results showed the AL/CS/DNA had a spherical structure. High DNA loading ability and effective DNA protection against nuclease were proved by gel electrophoresis. The surface charge of the AL/CS/DNA depended strongly on pH environment, enabling the intracellular release of loaded DNA via a pH-mediated manner. In comparison to the traditional CS/DNA system, our new design rendered a higher transfection efficiency and longer residence time of the AL/CS/DNA at nasal mucosal surface. These outstanding features enable the AL/CS/DNA to induce a significantly (*p*<0.01) higher level of secretory IgA (SIgA) than the CS/DNA in animal study, and a longer-term mucosal immunity. On the other hand, the AL/CS/DNA exhibited minimal cytotoxicity. These results suggest that the developed nanoparticles offer a potential platform for DNA vaccine packaging and delivery for more efficient elicitation of mucosal immunity.

## Introduction

The development of efficient and safe vaccines remains a major goal in globe public health. Recently, considerable attention has been focused on the development of DNA vaccines as an effective immunogenic strategy to induce both humoral and cellular immune responses. DNA vaccines are considered to be inexpensive, stable, relatively safe compared to attenuated viral vaccines [1], [2], [3], [4] and flexible to integrate genes encoding antigens and immunostimulatory sequences. Thus, DNA vaccines are in favor of worldwide transportation and vaccination. Though intramuscular (i.m.) vaccination is still widely used, unfortunately, it usually failed in inducing local immunity at mucosal sites where tremendous infectious agents enter the body [5]. Interestingly, nasal mucosa is an attractive site for DNA vaccines to evoke both systemic and mucosal immune response [6], [7], [8], [9], due to its accessibility, lower enzyme distribution compared to the oral route and dense population of immune cells, often referred to as the nasal associated lymphoid tissue (NALT).

It is believed that development of vaccine against *Streptococuus mutans* (*S. mutans*) might be an effective strategy to prevent and control the occurrence of dental caries. In previous studies [10], the DNA vaccine pGJA-P/VAX against dental caries has been successfully constructed by encoding a GLU domain of GTF enzymes along with the A and P regions of surface protein antigen (PAc) of *S. mutans*. The pGJA-P/VAX can induce specific saliva SIgA response and serum IgG response via i.n. or i.m. immunization in animals, including adult mice, rats, rabbits and monkeys [10], [11], [12]. In addition, the increase of the specific saliva SIgA is found to be correlated with reduction of dental caries [13], [14]. However, without adjuvants or delivery system, the single DNA vaccine (pGJA-P/VAX included) displayed low efficacy via i.n. administration [15], [16], possibly attributed to poor antigen delivery, as a result of various physiological hurdles, including mucociliary clearance, enzyme degradation and poor internalization by antigen presenting cells (APCs).

Nanoparticles (NPs) may provide an ideal solution to alleviate problems associated with the nasal delivery of DNA vaccines. Chitosan (CS) NPs are of special interest in nasal DNA vaccine delivery because of its unique properties, including bioadhesiveness, effectiveness in transport of antigens by transiently opening tight junctions of cells, biocompatibility and low toxicity [17], [18]. More evidences have shown that DNA vaccine complexed with the CS significantly improved mucosal sIgA levels via i.n. administration [19], [20]. As a DNA vaccine delivery system, the CS NPs are expected to increase the uptake and expression efficiency of the DNA vaccine in target cells. However, as compared to other non-viral carriers, the CS NPs mediated transfection efficiency is still relatively low [21], [22]. It has been suggested that the strength of electrostatic interactions between CS and DNA prevents their dissociation within cells, thus precluding transcription of DNA and resulting in low transfection [23].

To improve the transfection efficiency of CS/DNA complexes, additive incorporation of negative charged component to reach a balance between DNA protection and DNA release is an effective strategy [24]. It has been reported that a core-shell nanoparticle system comprised of an anionic PLGA core and a glycol CS/DNA shell surface has been shown for enabling intracellular release of the loaded DNA via a pH-mediated manner, so as to facilitate DNA vaccine transfection in target cells [25]. Anionic lipids, the natural component of eukaryotic cell membranes, have been shown to significantly increase the gene transfection efficiency in cell lines, even better than commercial cationic liposomes [26]. In addition to powerful membrane fusion ability, the anionic liposomes exhibit more advantages compared with the cationic liposomes, especially in cytotoxicity. It has been reported that cationic liposomes are extremely toxic to phagocytic cells, and thus should be used with caution *in vivo* [27], [28]. Interestingly, mucosal immunization with liposomes seems to be limited and especially dependent on co-administration of other co-stimulants, which is caused by the modulation interaction of liposomes with mucosa and the instability of liposomes under physiological conditions [29]. These results inspired us whether a new system comprised of the AL and CS/DNA could render the transfection efficiency, and further improve the mucosal immunity of DNA vaccine. To our knowledge, very little has been focused on the combined use of bioadhesive CS with AL in nasal DNA vaccine delivery.

In the present study, we used plasmid pGJA-P/VAX as our test DNA vaccine to prepare the CS/DNA and AL/CS/DNA NPs. We characterized the size and zeta potential of the two NPs under different pH conditions, as well as the morphology using transmission electron microscopy (TEM). The novel AL/CS/DNA delivery system exhibited both pH-mediated DNA release and enhanced mucoadhesive properties. The AL/CS/DNA was able to induce strong mucosal immune responses following i.n. immunization on Balb/c mice. These findings imply that our synthetic NPs present an appropriate nanoparticle delivery system to improve the mucosal immunogenicity of DNA vaccines by increasing antigen delivery, transport and stability along the mucosal tract.

## Materials and Methods

### 1. Materials

Chitosan (MW = 80 kD, degree of deacetylation = 80%) and mucin type III were purchased from Sigma (St. Louis, USA). Dioleoylphosphatidylethanolamine (DOPE) and 1, 2-Dioleoylphosphatidylglycerol (DOPG) were purchased from Lipoid (Ludwigshafen, Germany). Plasmid pGJA-P/VAX and recombination proteins PAc were provided by Prof. Mingwen Fan (School of Stomatology, Wuhan University, China). Plasmid pcDNA3.0-Rluc was donated by Dr. Sanjiv Sam Gambhir (Dept. of Radiology, Stanford University, USA). RQ1 DNase kit was purchased from Promega (Madison, MI, USA). Mouse standard serum (Bethyl, USA), goat anti-mouse IgG and IgA (SouthernBiotech, USA), and horseradish peroxidase conjugated goat anti-mouse IgG and IgA (SouthernBiotech, USA) were used. Cy5.5 Mono NHS Ester was purchased from GE Healthcare (Buckinghamshire, UK). All other used chemicals and used reagents were analytical grade.

### 2. Preparation of CS-based NPs

Anionic liposomes consist of DOPG/DOPE were prepared. Briefly, a lipid mixture at a molar ratio 1∶4 (DOPG:DOPE) was reconstituted in 10 mM HEPES buffer at pH 7.4 from shell-dried lipid films followed by vortexing for 10 min and sonication for 1 min. CS (5 mg/ml) was dissolved in 0.5% acetic acid overnight on a vortex mixer (IKA, Germany). Prior to mixing with DNA, CS solutions were subjected to sterile filtration with a 0.22 µm syringe filter. CS/DNA NPs were prepared by adding an equal volume of the diluted CS solution to a DNA (1000 µg/ml) solution at different N/P ratios (amine:phosphate = 3, 5, 7, 10, 15, mole ratio) at room temperature, using a vortex mixer. To prepare AL/CS/DNA NPs, anionic liposomes (DOPG/DOPE, 4 mg/ml) in 10 mM HEPES buffer were mixed with a DNA (1,000 µg/ml) solution at different lipid/DNA ratios (1, 2, 3, 4, w/w) to a final volume of 100 µl. CS solutions at different N/P ratios were then added dropwise under mild stirring. The pH values of the CS-based NPs were adjusted to 7.4, 7.1, 6.8 or 6.4 for characterization.

### 3. Characterization of CS-based NPs

The size distribution and the zeta potential of NPs were measured by dynamic light scattering (Nano ZS 90, Malvern, UK) at 25°C. NPs were diluted with PBS buffer at pH 7.4, 7.1, 6.8, 6.4, and stabilized at room temperature for 30 min prior to the measurement. The morphology of NPs was examined with TEM by the conventional negative staining method using 0.1% phosphotungstic acid. The release of pGJA-P/VAX from AL/CS/DNA in PBS was studied at different pH (7.4 or 6.4) in a shaker at 100 rpm and 37°C. The released free DNA was separated from NP by centrifugation at 14, 000 rpm for 20 min after different time intervals within 24 h and the concentration was quantified by using fluorescent dye Hoechst 33258. Each measurement was carried out in triplicate.

### 4. Protection Against Nuclease Degradation

The NPs were incubated with DNase I in reaction buffer at 37°C for 30 min, followed by the addition of DNase stop solution to terminate the reaction and DNase I was inactivated at 65°C for 10 min. Samples were analyzed by 0.8% agarose gel containing 1 µg/ml ethidium bromide in TAE buffer. The DNA was detected under UV light using the Gel Documentation System (Biorad, USA).

### 5. Adsorption of Mucin on CS-based NPs

Equal volumes of the CS-based NPs and an aqueous solution of mucin (1 mg/ml) were mixed by vortexing for 60 min. The suspension was then centrifuged at 4,000 rpm for 5 min. The supernatant was mixed with 0.5% periodic acid and incubated at 37°C for 2 h. A colorimetric assay for glycoproteins based on the periodic acid/Schiff staining was used for determination of mucin concentration in the supernatant [30]. The mucin adsorbed on the CS-based NPs was calculated from the total and the free mucin. Naked DNA was used as the control.

### 6. *In vitro* Transfection

The human embryonic kidney cells (HEK 293, ATCC, USA) were cultured in DMEM supplemented with 10% fetal bovine serum (FBS) at 37°C in a humidified 5% CO_2_ incubator (Thermo, USA). Cells were subcultured according to ATCC recommendations without any antibiotics. Cells were seeded into a 24-well plate with a density of 2 × 10^5^ cells per well. Transfection proceeded until approximately 60–80% confluence was achieved. The transfection medium DMEM without FBS was equilibrated at 37°C with 5% CO_2_, and pH adjustment was performed with sterile acetic acid. CS/Luc and AL/CS/Luc NPs containing plasmid pcDNA3.0-Rluc were prepared (as described above) 30 min before transfection of cells. The medium was removed and replenished with 400 µl transfection medium (pH 6.4) containing CS/Luc or AL/CS/Luc at a concentration of 1 µg DNA/well. After 5 h, the transfection medium was removed and replaced with 500 µl DMEM (with 10% FBS). The cells were further incubated for 48 h, and then harvested by using trypsin solution. Transfection efficiencies were quantified with luciferase assay kit (Promega, USA). PEI (25 kD, Sigma, USA) was used as a positive control. The PEI/Luc complexes were prepared by directly mixing at the N/P ratio of 10. The relative light units (RLU) were normalized to the protein content of each sample. All experiments were repeated at least three times.

### 7. Transport of NPs Across the Nasal Epithelia

FITC-CS/DNA and AL/FITC-CS/DNA NPs were prepared and administrated into rat nasal cavity with 10 µl into each nostril within 10 min interval. After administration for 2 h, rats were sacrificed by cervical dislocation. 4% paraformaldehyde solution was injected to nasal cavity from trachea with a syringe. After fixing for 30 min, the rat mucosa was carefully excised and observed by Confocal Laser Scanning Microscope (Andor Revolution XD).

### 8. Transepithelial Electrical Resistance (TEER) Detection

Caco-2 cells (ATCC, USA) were cultured in DMEM supplemented with 10% FBS, 4.5 g/L glucose, 2 mM L-glutamine, 1% v/v nonessential amino acids and 500 U/ml penicillin/streptomycin at 37°C and 5% CO2. For TEER detection experiments, the Caco-2 cells were seeded in 24-well Transwell (2 × 10^5^ per insert) and maintained for 7 days in supplemented DMEM. Medium was changed every day. The integrity of the monolayer was assayed by measuring the TEER using an electrode connected to EVOM^2^ (WPI, USA). Then Caco-2 monolayers were washed once with Hanks balanced salt solution (HBSS, pH 6.4) and equilibrated in HBSS for 30 min at 37°C. After treating with DNA, CS/DNA and AL/CS/DNA in HBSS for 1 h, inserts were transferred to a 24-well plate containing 1.5 ml HBSS and TEER was assessed. All experiments were repeated at least three times.

### 9. Uptake of CS-based NPs by Flow Cytometry

FITC-CS was synthesized by the reaction between the isothiocyanate group of FITC and the amino group of CS. For detecting the uptake of FITC-CS/DNA and AL/FITC-CS/DNA, Caco-2 cells were plated at 2 × 10^6^ cells/well in 6-well plates and allowed to adhere overnight. After treating with FITC-CS/DNA or AL/FITC-CS/DNA at a concentration of 4 µg DNA/well, cells were harvested and suspended in PBS and analyzed directly by flow cytometry. Samples were run on a Cytomics™ FC 500 flow cytometer (Beckman Coulter, USA) and analysis was done using CXP software.

### 10. Immunization Studies and Sample Collection

The ability of pGJA-P/VAX complexed with or without CS-based NPs to induce immune response was evaluated in female Balb/c mice (aged 6–8 weeks). The animals were housed in groups of five (n = 10) with free access to food and water in a specific pathogen-free environment at the Hubei Medical Laboratory Animal Center (Wuhan, China). The studies were carried out according to the guidelines provided by the Hubei Medical Laboratory Animal Center review board. The protocol was approved by the Committee on the Ethics of Animal Experiments of Huazhong University of Science and Technology (Permit Number: SCXK 2008-0005). All surgery and was performed under sodium pentobarbital anesthesia, and all efforts were made to minimize suffering. To evaluate the immune response, five groups with different administration routes were performed as follows: naked DNA (i.n.), naked DNA (i.m.), CS/DNA (i.n.), AL/CS/DNA (i.n.) and PBS group (i.n.). Every animal was inoculated with 25 µg plasmid pGJA-P/VAX in small drops. Nasal dosing was carried out with 10 µl sterile micropipette tips and given into the nostril (10 µl formulation/nostril) of the non-anesthetized animal in a supine position. The new drop was only given when the former one had been inhaled. Animals were also immunized with 25 µg plasmid pGJA-P/VAX in HEPES (pH 7.4) via i.m. pathway (in quadriceps muscles). A secondary immunization was performed after 2 weeks with the same dose of formulations and operating procedures. Saliva and serum were collected at 2, 4, 6, 8, and 12 weeks after the last immunization, respectively. Each animal was intraperitoneally injected with 100 µl sterile pilocarpine solution (10 mg/ml) in order to collect saliva samples. These fluids were stored at −70°C with 100 mM phenylmethyl sulfonyl fluoride (PMSF) as a protease inhibitor. Blood was collected by retro-orbital puncture with a syringe, and serum was then obtained and stored at −70°C after centrifugation.

### 11. Measurement of Specific IgA and IgG Responses

The levels of specific antibodies against PAc in the samples were determined by an enzyme-linked immunosorbent assay (ELISA). Each well of the 96-well plates (Costar USA) was coated with purified 10 µg/ml protein PAc in a 0.1 M, pH 9.5 carbonate buffer at 4°C. After coating overnight, plates were blocked by 3% bovine serum albumin (BSA) in PBS containing 0.05% Tween 20 (PBST) at 37°C for 2 h, then washed five times with the PBST. Diluted saliva (1∶2 dilution) or serum (1∶100 dilution) was added into each well and incubated for 30 min at 37°C. A standard curve was established by coating plates with unconjugated goat anti-mouse IgG or IgA (10 µg/ml). Then, the serially diluted reference serum, used to create a standard curve, was added in duplicate into each well and incubated at 37°C for 2 h. Subsequently, the plates were washed five times with PBST and reacted with peroxidase-conjugated goat anti-mouse IgG (dilution 1∶1000) or peroxidase-conjugated goat anti-mouse IgA (dilution 1∶1000) in PBST. After washing, plates were followed by adding TMB substrate with H_2_O_2_ and incubation at 37°C for 15 min. Then the reaction was stopped with 2 M H_2_SO_4_. Optical density (OD) readings were taken and recorded at 450 nm. The sample specific antibody concentrations were calculated based on multi-parameter logistic algorithms and the final results were counted according to the standard curve.

### 12. *In vivo* Fluorescence Imaging

The NIRF dye Cy5.5 was modified to the amino group of CS for real-time imaging *in vivo.* The nasal residence time measurements were performed according to the protocol described by Hagenaars et al [31]. Briefly, female Balb/c (nu/nu) mice aged 6 weeks from Hubei Medical Laboratory Animal Center (Wuhan, China) were anesthetized with isoflurane prior to the administration of 10 µl AL/Cy5.5-CS/DNA NPs. After wiping the nose with a paper towel, mice were immediately scanned with the IVIS spectrum imaging system (Caliper life, Cy5.5 filter). Between the measurements, mice were put back in cages to recover from anesthesia. In order to calculate the mean fluorescence in the nasal cavity, the Cy5.5 specific signal was separated from the background fluorescence by spectral unmixing with the Living Imaging 4.0 software (Caliper life Science). Regions of interest (ROI) in the nasal cavity were detected and quantified with the same software. Fluorescence emission images were normalized and reported as photons per second per centimeter squared per steradian (p/s/cm^2^/sr). Fluorescence intensity at t = 0 was set as at 100%.

### 13. Statistical Analysis

Statistical analysis of specific antibody levels was performed with SPSS 17.0 software. Differences between different groups were determined by one-way analysis of variance (ANOVA) followed by post hoc analysis with LSD test. Comparisons were deemed significant when *p*<0.05 and highly significant when *p*<0.01.

## Results

### 1. DNA Loading and Nuclease Degradation Assay

**Figure 1:**
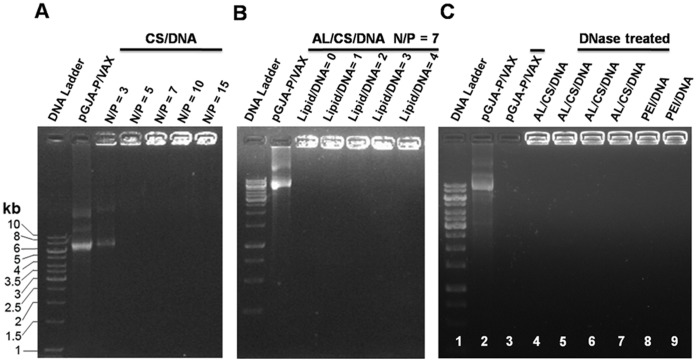
Gel retardation analysis of CS-based NPs using plasmid pGJA-p/VAX as the DNA model. (A) CS/DNA, at different N/P ratios. (B) AL/CS/DNA, at different lipid/DNA (w/w) ratios with a fixed N/P ratio of 7. (C) AL/CS/DNA, treated with DNase I at the lipid/DNA (w/w) ratio of 3 with a fixed N/P ratio of 7. lane 1: DNA ladder; lane 2: pGJA-p/VAX; lane 3: pGJA-p/VAX+DNase I (50 U/ml); lane 4: AL/CS/DNA; lane 5: AL/CS/DNA+DNase I (50 U/ml); lane 6: AL/CS/DNA+DNase I (100 U/ml); lane 7: AL/CS/DNA+DNase I (200 U/ml); lane 8: PEI/DNA+DNase I (200 U/ml); lane 9: PEI/DNA.

Gel electrophoresis has been widely used to monitor the affinity capability of DNA adsorbed on test NPs. As shown in Fig. 1A, pGJA-P/VAX was completely compacted by CS as the N/P ratio reached 7. In contrast, partial dissociation of pGJA-P/VAX from the CS/DNA was observed at the N/P ratio of 3. Although no obvious migration of DNA was detected from the CS/DNA at the N/P ratio of 5, less DNA was retarded compared with higher N/P ratio NPs. Therefore, the CS/DNA was prepared at the N/P ratio of 7 in the subsequent experiments. As shown in Fig. 1B, no significant DNA release was observed by incorporating AL in the CS/DNA at various lipid/DNA ratios (w/w) with fixed N/P ratio of 7. The influence of AL incorporation on the size, PDI, zeta potential and encapsulation efficiency was summarized in Table 1. With increased amount of AL incorporated, size of ALCS/DNA increased while their PDI decreased notably. In addition, modification CS with FITC or Cy5.5 did not interfere with DNA encapsulation.

**Table 1:** Physicochemical characteristics of CS/DNA and AL/CS/DNA.

Formulations (N/P, lipid/DNA)	Size (nm)	PDI	Zeta potential (mV)	Encapsulation efficiency (%)
CS/DNA (7, 0)	323.5±20.3	0.259±0.014	4.8±1.3	96.46±2.41
AL/CS/DNA (7, 1)	312.7±14.7	0.205±0.082	4.5±0.7	95.32±0.48
AL/CS/DNA (7, 2)	320.1±12.3	0.214±0.054	8.1±0.8	97.34±1.02
AL/CS/DNA (7, 3)	319.5±16.9	0.134±0.027	6.7±0.3	96.36±1.35
AL/CS/DNA (7, 4)	498.5±28.9	0.125±0.032	2.5±0.8	95.26±0.45
FITC-CS/DNA (7, 0)	340.5±27.4	0.237±0.044	4.4±0.5	99.42±0.11
AL/FITC-CS/DNA (7, 3)	338.5±12.1	0.101±0.025	7.7±0.2	98.24±0.21
Cy5.5-CS/DNA (7, 0)	328.6±15.6	0.233±0.036	4.0±1.1	96.64±0.25
AL/Cy5.5-CS/DNA (7, 3)	315.4±18.1	0.128±0.035	6.1±1.6	96.24±0.34

Particle size, polydispersity index (PDI), zeta potential and encapsulation efficiency of tested NPs with different N/P ratios and lipid/DNA ratios under pH 6.4 (n = 3).

In Fig. 1C, the AL/CS/DNA effectively protected DNA against degradation of DNase I, whereas the naked DNA was easily digested by low concentration of DNase I (50 U/ml). PEI/DNA was also shown to have high DNA loading and high resistant against DNase.

These results indicated that the AL/CS/DNA could be successfully prepared with high DNA loading and high DNase resistance. The high enough electrostatic interaction between the CS and the DNA might be responsible for effective DNA loading, and presumably cause the inability of enzyme to reach the substrate DNA. At the physiological condition of mucosal membrane surfaces, where large quantities but low concentration of nucleases present, it would be meaningful for maintaining the integration and function of DNA vaccine.

### 2. *In vitro* Transfection Assay

**Figure 2:**
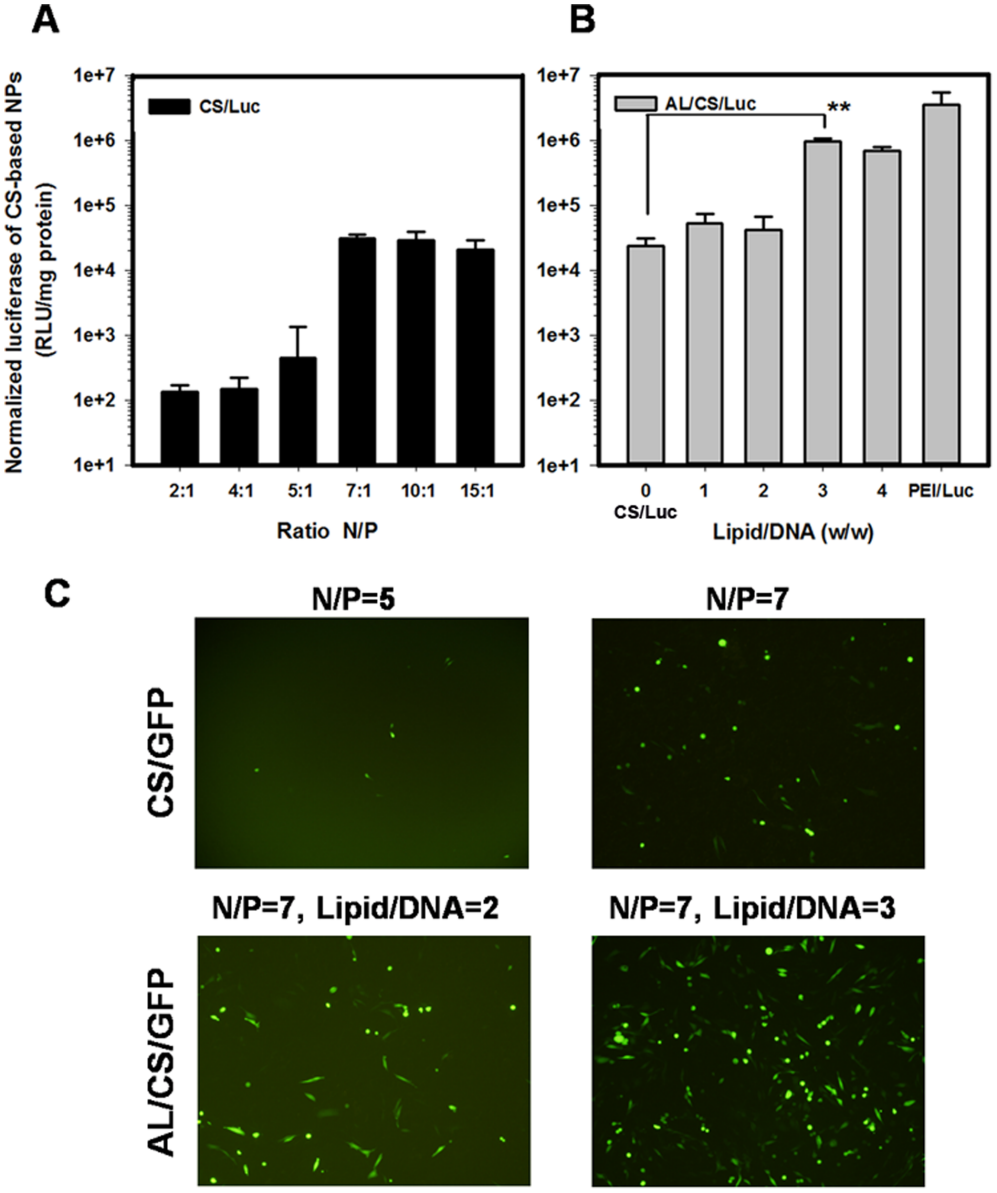
Transgene expression by HEK 293 cells in vitro using CS/DNA, AL/CS/DNA and PEI/DNA NPs. (A) CS/Luc, at different N/P ratios with fixed 1 µg plasmid pcDNA3.0-Rluc/well and fixed transfection medium pH of 6.4; (B) AL/CS/Luc, at different lipid/DNA ratios (0, 1, 2, 3, 4, w/w) with fixed N/P ratio of 7 and fixed transfection medium pH of 6.4 and 1 µg plasmid pcDNA3.0-Rluc/well. PEI/Luc NPs at N/P ratio of 10 were used as the positive control; (C) Transfection images of CS/GFP and AL/CS/GFP NPs with different N/P ratios and lipid/DNA ratios using GFP as a report gene. The relative light units (RLU) were normalized to the protein content of each sample. **p<0.01 Mean ± SD (n = 4–6).

Furtherly, transfection assay was performed to confirm the optimal N/P ratio and lipid/DNA ratio, which might contribute to high expression of DNA vaccine in target cells. HEK 293 cells were used as the model for evaluating the transfection efficiencies by the expression of luciferase reporter enzymes and GFP (Green Fluorescence Protein). As shown in Fig. 2A, transfection efficiency of the CS/Luc at different N/P ratios was determined by luciferase assay at pH 6.4. The results indicated that the optimal N/P ratio was from 7∶1 to 10∶1, which agreed with the previous results [32]. Transfection efficiency of the AL/CS/Luc was optimized under various lipid/DNA ratios (w/w). PEI/Luc NPs were used as the positive control (Fig. 2B). These results showed that the transfection efficiency of AL/CS/Luc was notably enhanced as lipid/DNA ratio (w/w) increased to 3. Transfection images of CS/GFP and AL/CS/GFP with different N/P ratios and lipid/DNA ratios were shown in Fig. 2C. Obvious better transfection results were verified on the AL/CS/DNA than that of the CS/DNA. Finally, the optimal AL/CS/DNA was prepared at N/P ratio of 7 and lipid/DNA ratios (w/w) ratio of 3.

### 3. Characterization of CS-based NPs

**Figure 3:**
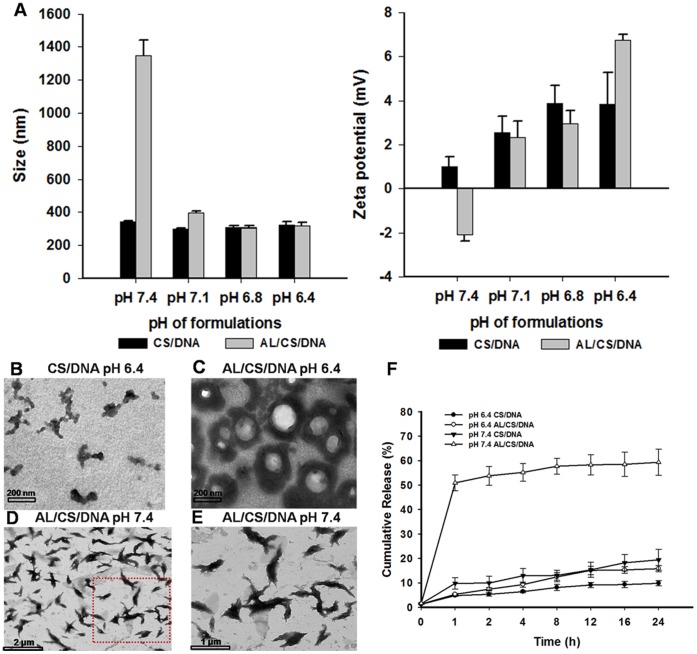
Characterization of the CS/DNA and AL/CS/DNA NPs. (A) Size and zeta potential of CS/DNA and AL/CS/DNA at pH 7.4, 7.1, 6.8 and 6.4. (B) TEM images of the CS/DNA at pH 6.4; (C) TEM images of the AL/CS/DNA at pH 6.4. (D) TEM images of the AL/CS/DNA at pH 7.4; (E) Local amplification of (D); (F) Release profile of DNA from AL/CS/DNA and CS/DNA in PBS (pH 6.4 or 7.4) at 37°C. Mean ± SD (n = 3).

The physicochemical properties of NPs influence their stability, mucosal adsorption and transport, which are the three indispensable steps for the successful mucosal immunity. After intranasal administration, the NPs might be challenged by pH environment changes *in vivo*. Thus, the influence of pH change on the CS/DNA and AL/CS/DNA was examined. As shown in Fig. 3A, the AL/CS/DNA was found to have a significant change in size and zeta potential, especially when the pH increased to 7.4 from 7.1. The size sharply increased to micron scale and the zeta potential decreased to −2.1 mV, indicating the disintegration of the ternary complex structure. However, the size and zeta potential of the CS/DNA did not change significantly. Studies on the surface morphology of the prepared NPs with TEM indicated that the CS/DNA was irregular shape (Fig. 3B), while the AL/CS/DNA was almost spherical at pH 6.4 and AL acted as a core (Fig. 3C). When pH increased to 7.4, the disintegration of the AL/CS/DNA was observed (Fig. 3D and 3E). As we expected, a pH-mediated DNA release from the AL/CS/DNA was verified in Fig. 3F. These results indicated that with the addition of AL in CS/DNA, homogeneous AL/CS/DNA NPs were formed. In addition, the AL might facilitate the DNA release from the AL/CS/DNA with a pH-mediated manner.

### 4. Nasal Residence Time and Mucin Adsorption Ability of CS-based NPs

**Figure 4:**
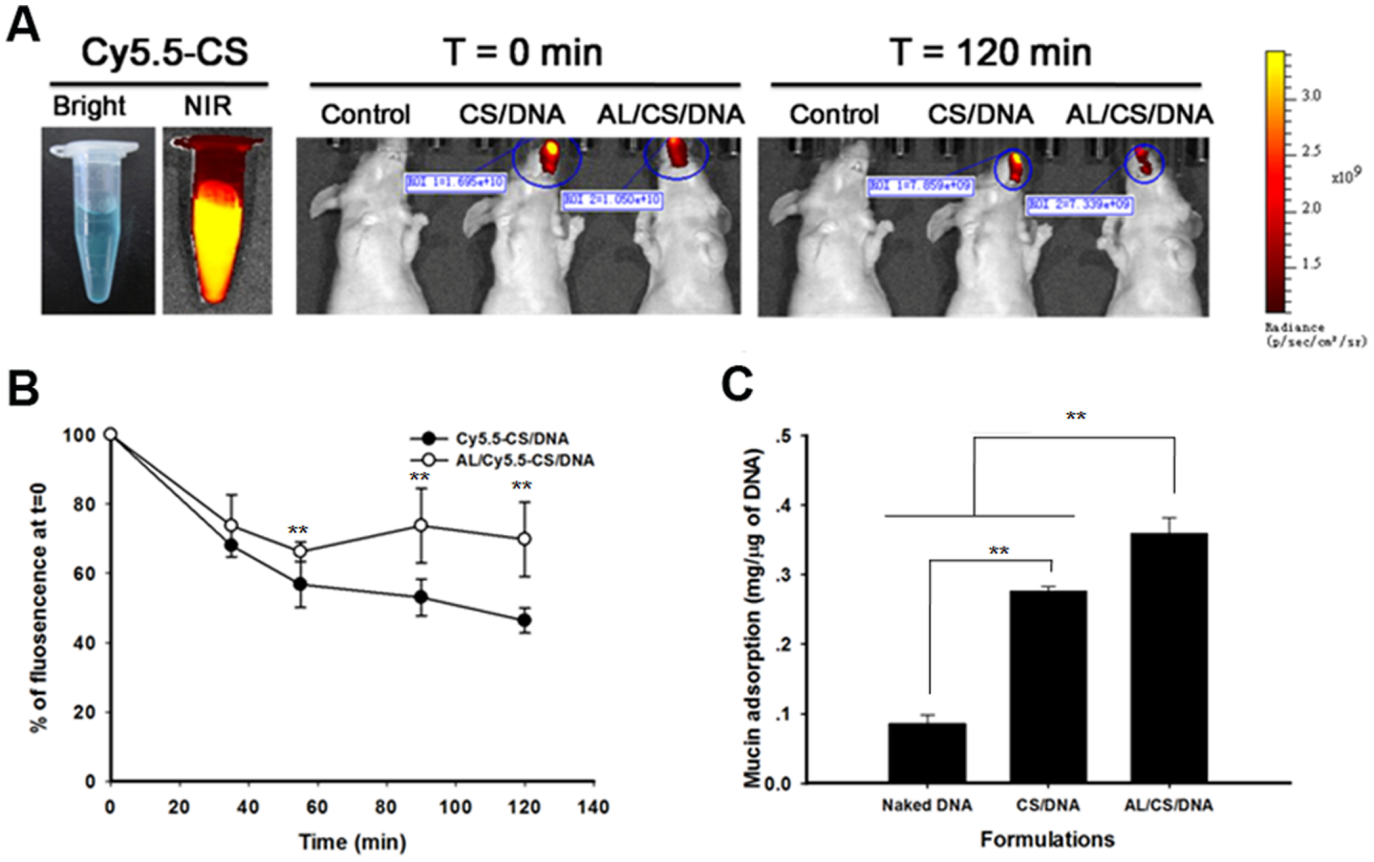
Analysis of nasal residence time and mucin adsorption ability of CS-based NPs. (A) Fluorescence images of nasal clearance of Cy5.5-CS/DNA and AL/Cy5.5-CS/DNA after i.n. administration. (B) Clearance derived from spectra. Fluorescence at t = 0 was set as 100%. (C) Adsorption of mucin on the CS/DNA and AL/CS/DNA. Formulations were prepared at pH 6.4; **p<0.01 Mean ± SD (n = 4).

It is being assumed that increasing nasal residence time of antigen is one of the key features to enhance the nasal immune responses. The nasal residence time of Cy5.5-CS/DNA and AL/Cy5.5-CS/DNA were monitored with IVIS spectrum imaging system as in Fig. 4A. We found that the fluorescence of the two NPs in the cavity exceeded 40% of total fluorescence after i.n. administration within 2 h (Fig. 4B). However, in comparison to the CS/DNA, a more powerful ability in prolonging the nasal residence time was verified on the AL/CS/DNA.

The CS/DNA and AL/CS/DNA were also evaluated for their mucin adsorption ability, which is a measure of mucoadhesive ability of delivery system (Fig. 4C). The CS/DNA was found to retain more mucin (0.27 mg/µg of DNA) than the naked DNA (0.08 mg/µg of DNA) (*p*<0.01). The AL/CS/DNA had the more powerful adhesive ability than the CS/DNA, since the mucin adsorption reached 0.36 mg/µg of DNA. However, the increased fold was less than 1.5. These results indicated that addition of AL to the CS/DNA complexes increased the nasal residence time of DNA vaccine on mucosal surface. The prolonged residence time in nasal cavity offered a sustained antigen-mucosa interaction without being cleared away.

### 5. Transport of NPs in Rat Nasal Mucosa

**Figure 5:**
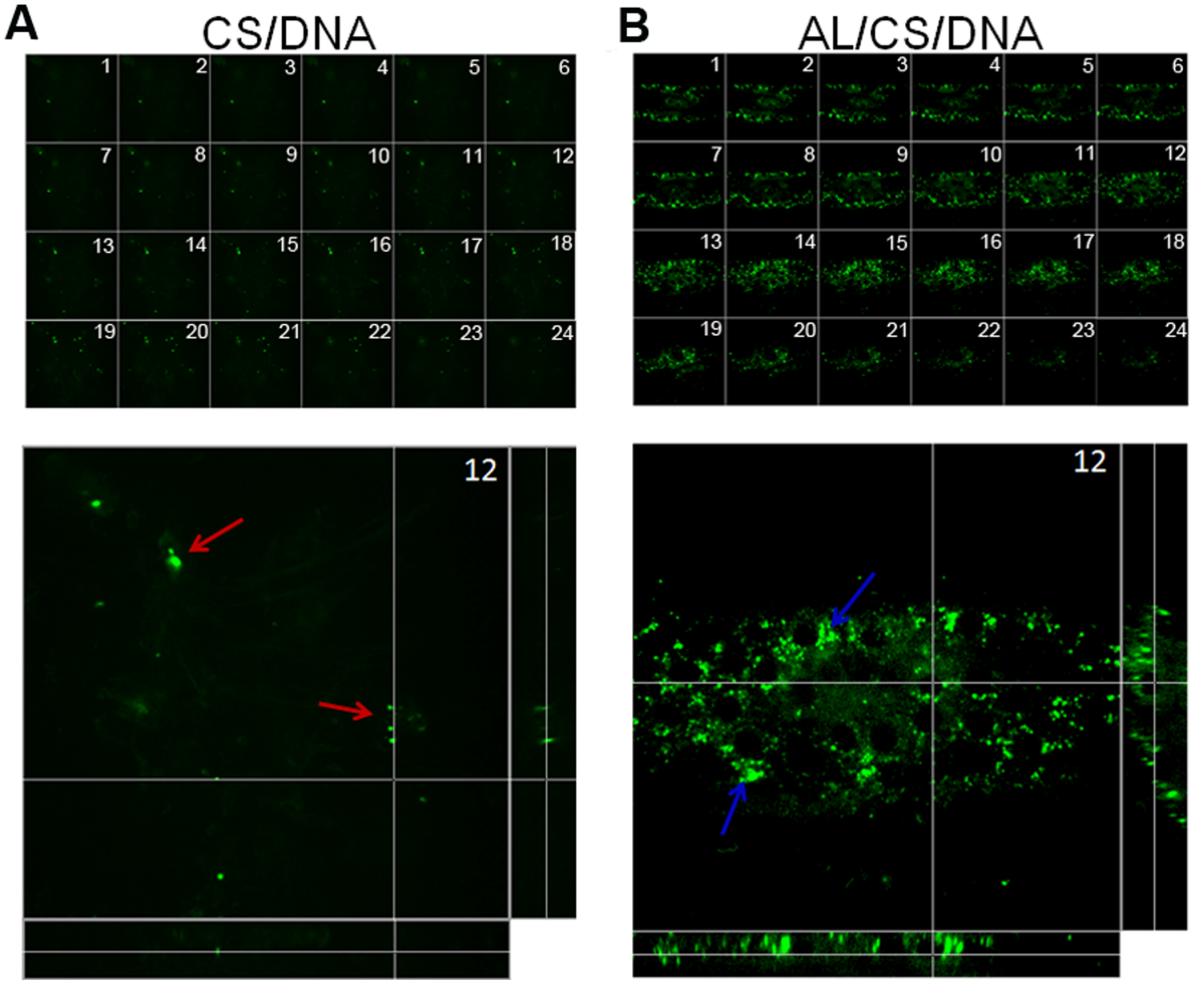
Representative Z-scan images of the FITC-CS/DNA and AL/FITC-CS/DNA treated rat nasal mucosa. (A) FITC-CS/DNA, treated for 2 h. (B) AL/FITC-CS/DNA, treated for 2 h. Both the 24 images were from the apical side to depth of 17.6 µm beneath mucosa and in successive steps with 0.8 µm apart. Images of the x, z and y, z sections captured in the depth of 8.8 µm inside mucosa from the apical side were shown below. Red arrow represents FITC-CS/DNA; Blue arrow represents AL/FITC-CS/DNA.

In order to investigate the transport of NPs in the nasal mucosa, FITC-CS/DNA and AL/FITC-CS/DNA were administrated into rat nasal mucosa for 2 h, and the transport of NPs was monitored by CLMS. It is proved that FITC modification on CS did not interfere with DNA entrapping capacity and release (Table 1 and Fig. S1). As shown in Fig. 5B, the AL/FITC-CS/DNA was found to penetrate into the deep mucosa layer (up to 17.6 µm) within 2 h. The fluorescence intensity increased to the peak value in the depth of 8.8 µm and lasted to the depth of 13.6 µm, then decreased gradually in the deeper depth. The images of the sections (x, z and y, z) taken in the depth of 8.8 µm inside the mucosa were also shown below. It clearly showed that the AL/FITC-CS/DNA were internalized into mucosal epithelia and transported into deeper regions of mucosal area. However, as shown in Fig. 5A, less FITC-CS/DNA was found to penetrate into the mucosa layer. The three dimensional reconstruction animations from CLMS results were shown in Movie S1 and Movie S2. These results provided the evidence that the AL/CS/DNA was more powerful in penetrating the mucosa layer.

### 6. TEER Change and Uptake of CS/DNA and AL/CS/DNA on Caco-2 Cells

**Figure 6:**
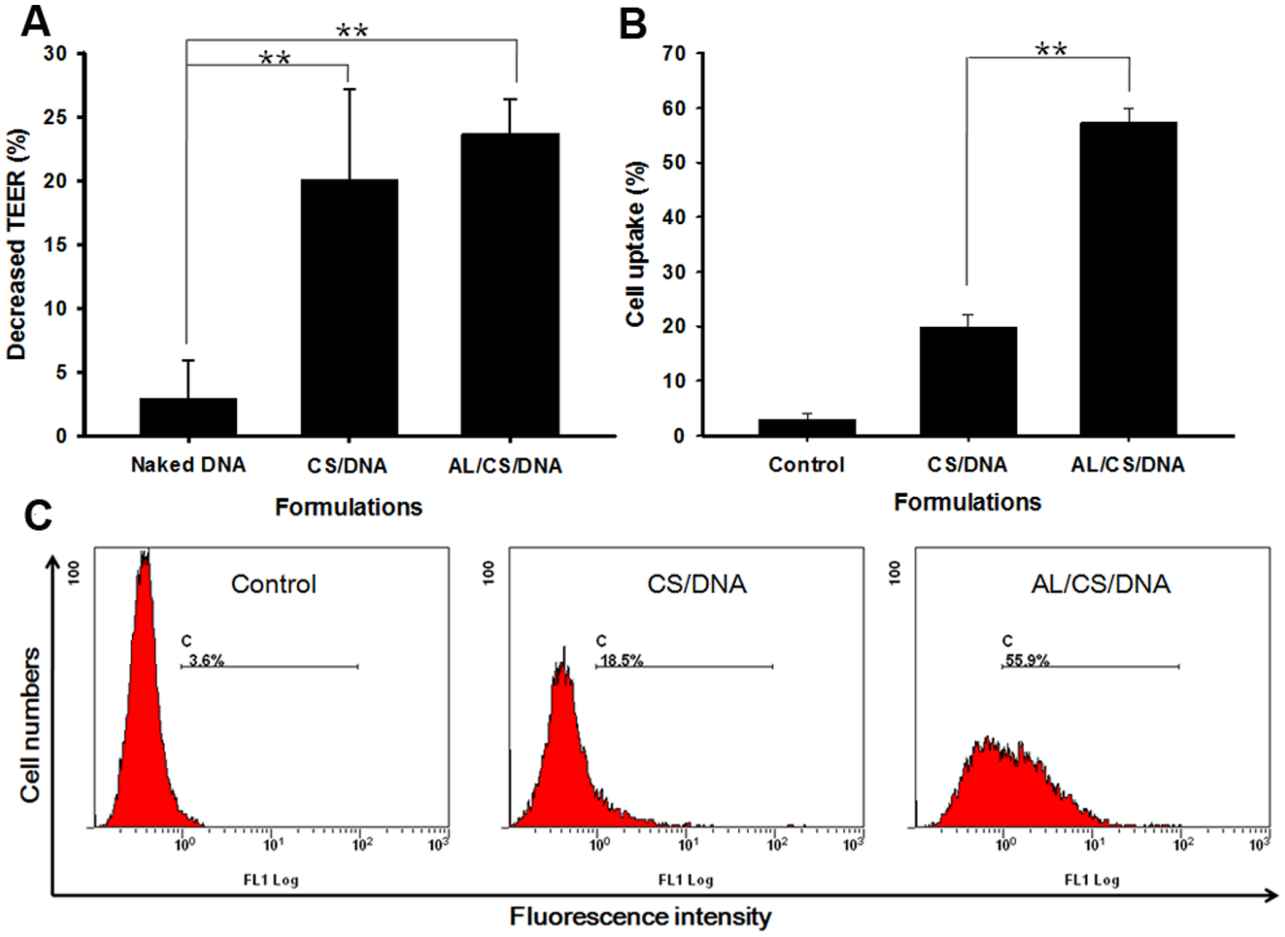
TEER decrease and intracellular uptake on Caco-2 cells after CS/DNA and AL/CS/DNA treatment. (A) TEER decrease of Caco-2 cell monolayer after 1 h exposure to the above formulations. (B) Percentages of intracellular uptake of test formulations after administration for 2 h. (C) Flow cytometry pictures are representatives of each group. (n = 3) *p<0.05, **p<0.01.

To better understand the mechanism leading to the more powerful transport of AL/CS/DNA in nasal mucosa, Caco-2 cells were used as the model to investigate the mucosa permeability and the intracellular uptake. TEER decrease on Caco-2 cell monolayer is a measure for mucosal epithelial permeability. Interestingly, although both CS/DNA and AL/CS/DNA showed the power in decreasing the TEER, there was no obvious difference between the two NPs (Fig. 6A). Then, the percentage of cells that internalized FITC-labeled NPs and their fluorescence intensity were quantified by flow cytometry. As shown in Fig. 6B and 6C, the percentage of fluorescent cells and the fluorescence intensity were enhanced notably (p<0.01) by incorporating AL. These results indicated that incorporating AL in CS/DNA might increase the intracellular uptake in epithelial cells without affecting the inherent mucosal permeability.

### 7. Immunization Studies

**Figure 7:**
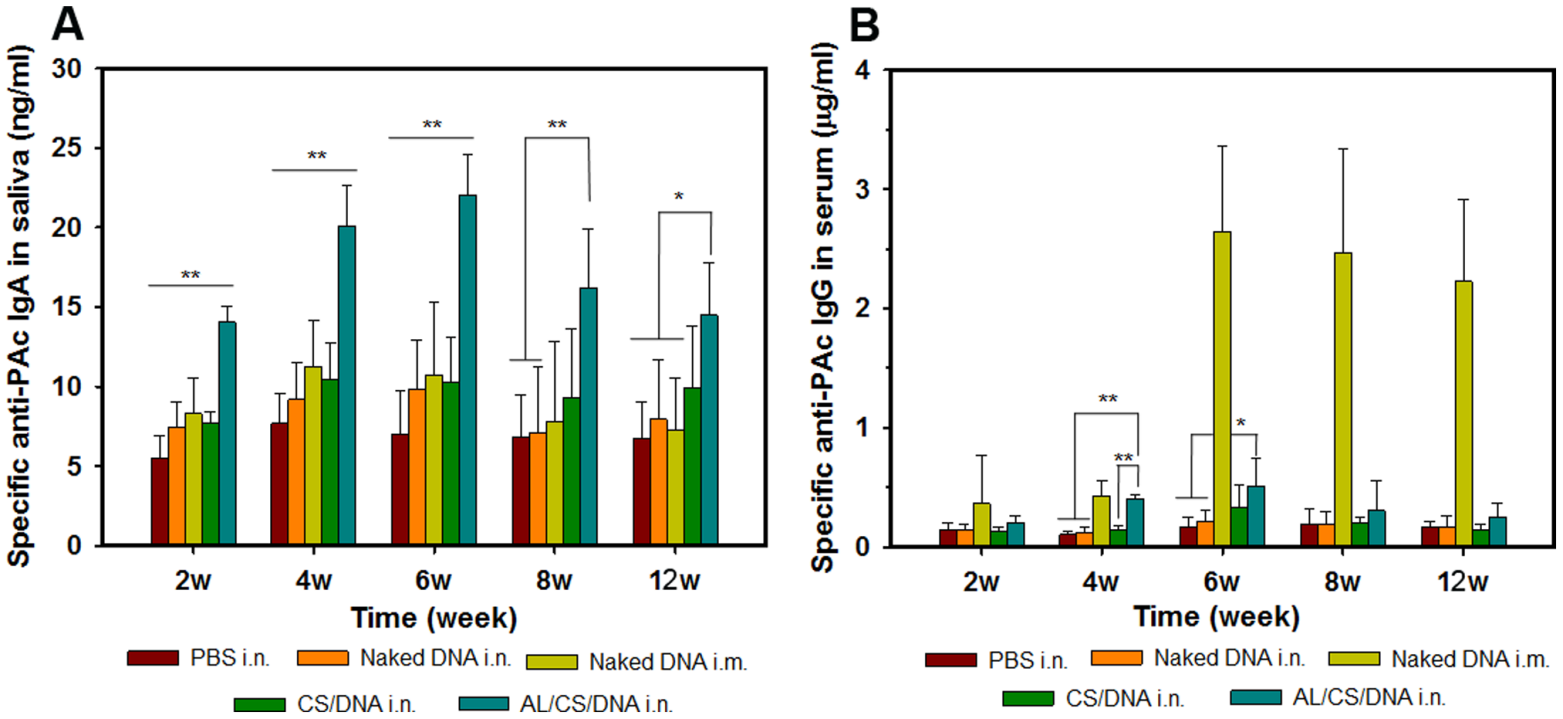
Antibody levels of saliva and serum collected from mice immunized with different formulations and routes. (A) Specific anti-PAc salivary IgA levels (ng/ml); (B) Specific anti-PAc serum IgG levels (µg/ml). Samples were collected at weeks 2, 4, 6, 8 and 12. Compared with the AL/CS/DNA (i.n.) group, *p<0.05, **p<0.01; Mean ± SD (n = 10).

In order to evaluate the potential of the pGJA-P/VAX loaded CS-based NPs in inducing specific mucosal and systemic humoral responses, female Balb/c mice were divided into five groups (n = 10) and vaccinated with indicated formulations via i.n. or i.m. route. We assessed the specific anti-PAc antibody levels both in salivary and serum samples. As in Fig. 7A, a significant and lasting salivary anti-PAc SIgA antibody response was elicited by i.n. administration of the AL/CS/DNA. The mucosal immunity was induced in AL/CS/DNA (i.n.) group as early as 2 weeks after the first vaccination. It was significantly higher than the levels of naked DNA group and CS/DNA group (*p*<0.01). This immune effect was further strengthened to week 6, and then slightly decreased at week 8 and 12. In contrast, there was no significant difference in SIgA production between the CS/DNA (i.n.) group and the naked DNA (i.n.) group in all time intervals. In addition, a long-term mucosal immunity was found to last for at least 12 weeks in the AL/CS/DNA (i.n.) group. Week SIgA response was observed on the naked DNA (i.n.) group and the naked DNA (i.m.) group, indicating that DNA without delivery system did not seem to trigger effective SIgA responses via i.n. or i.m. route.

When mice were vaccinated with 25 µg pGJA-P/VAX via i.m. administration, strong serum response last at least for 12 weeks (Fig. 7B), which was in accordance with majority of previous studies showing successful DNA immunization [33], [34]. No obvious specific IgG response was observed in the PBS group (i.n.), naked DNA (i.n.) group or the CS/DNA (i.n.) group. In addition, specific IgG response of the AL/CS/DNA (i.n.) group presented statistical significance only at week 4 (*p*<0.01) compared with the CS/DNA (i.n.) group. These results indicated that the AL/CS/DNA was an attractive mucosal DNA vaccine delivery system for inducing strong mucosal responses. However, the type and intensity of immune responses were greatly affected by the administration routes.

### 8. Cytotoxicity Assay

**Figure 8:**
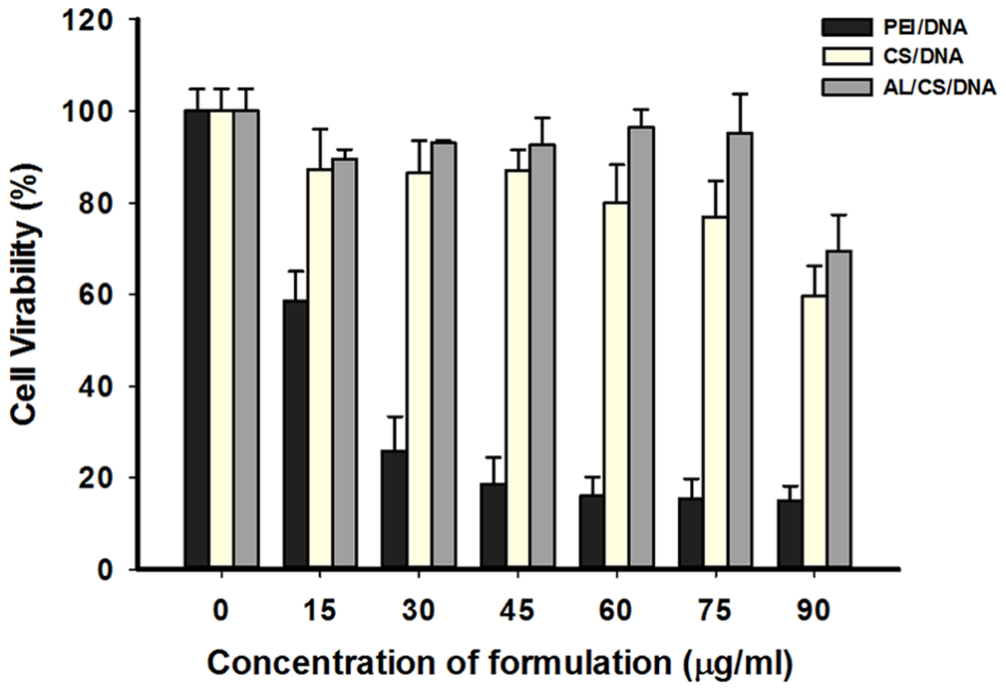
MTT results of the RAW 264.7 cells viability after treating with indicated concentrations of previously prepared PEI/DNA, CS/DNA and AL/CS/DNA NPs. Mean ± SD (n = 4).

As a vaccine delivery system, the potential cytotoxicity should be taken into serious consideration. The cytotoxicity of the PEI/DNA, CS/DNA and AL/CS/DNA was performed by MTT assay following 48 h incubation on RAW 264.7 cells (Fig. 8). The CS/DNA and AL/CS/DNA showed similar and high cell viability under varying concentrations. More than 80% cell viability was observed on the CS/DNA and AL/CS/DNA group under a concentration of 45 µg/ml, while the PEI/DNA showed high cytotoxicity even at the low concentration of 15 µg/ml. As the concentration reached 45 µg/ml, the cell viability of the PEI/DNA was less than 20%. These results indicated that the AL/CS/DNA had minimal cytotoxicity towards phagocytic cells.

## Discussion

It is believed that development of vaccine against *S. mutans* might be an effective strategy to prevent and control the occurrence of dental caries. Though i.n. delivery of pGJA-P/VAX-bupivacaine induced both mucosal humoral and systemic responses in various animals (mice, rats and monkeys), bupivacaine was not safe when used as an immune adjuvant. Meanwhile, the single pGJA-P/VAX seemed to work inefficiently via i.n. administration [16], [35]. In the present study, we demonstrated that after the incorporation of AL with CS/pGJA-P/VAX, enhanced cell uptake, cell transfection and mucoadhesive properties were observed. Finally, the AL/CS/DNA was found to induce strong mucosal and relatively weak systemic antibody responses following i.n. immunization in Balb/c mice.

The pKa values of CS and DOPG are 6.5 and 3, respectively [36], [37]. When formulations were prepared in water at pH 5.5, the ionized CS, DNA and DOPG can form ternary complexes (AL/CS/DNA) by electrostatic interactions between the positively charged amino groups on CS and the negatively charged phosphate groups on DNA and DOPG. TEM results revealed that the AL/CS/DNA had a relatively spherical shape and an AL core at pH 6.4 (Fig. 3C). Theoretically, when CS, DNA and AL are mixed together, two domains (CS/DNA and CS/AL) are formed because of electrostatic interactions. Thus, it is more likely that the DNA, AL and CS separately yielded two types of domains (CS/DNA and CS/AL), which were bridged by CS chains in the AL/CS/DNA NPs (Fig. S2). Similar two-domain structure has been reported by incorporation of poly(g-glutamic acid) in CS/DNA complex [24]. The AL/CS/DNA had a micron scale size and negative zeta potential when pH increased to 7.4 from 6.4 (Fig. 3A). In this process, most CS amine groups were deprotonated and CS became hydrophobic. As the electrostatic interactions were no longer significant, hydrogen bonding might be the driving force for CS/DNA binding and CS/AL binding. As both CS/DNA and CS/AL carried negative charges, the electrostatic repulsion between the two resulted in disintegration of the AL/CS/DNA structure and favored the release of loaded DNA. This might explain why the DNA release from the AL/CS/DNA was pH-dependent (Fig. 3F). In contrast to AL/CS/DNA, it was reported that the binding of CS and DNA was even tighter for CS/DNA complexes when pH increased to physiological environment and CS/DNA complexes were not disrupted [38].

Noticing the pH environments in the cytoplasm and nuclei of cells (pH 7.2–7.6), it can be inferred that after internalization into cells, the AL/CS/DNA could rapidly release a significant amount of the loaded DNA and facilitate expression of the encoded proteins. It is reported that the nuclear translocation and gene expression are not achieved unless DNA is released from complexes once inside cells [39]. Another reason might be responsible for the high tranfection efficiency of the AL/CS/DNA. DOPE (one component of AL) were reported to promote membrane destabilization and suggested to facilitate gene transfection [40]. The PG lipids were proved to have nucleus-fusogenic ability [41], which might facilitate the nuclear translocation of DNA. In addition, the nasal mucosal pH is approximately 5.5–6.5 [42], which is in favor of stabilizing the AL/CS/DNA without DNA release before internalization. Excitingly, enhanced gene expression in nasal mucosa was observed (Fig. S3), which implied that the AL/CS/DNA might be more powerful than CS/DNA when used for nasal mucosa vaccination. In comparision, no fluorescence signal was detected when naked DNA was used.

Interestingly, the AL/CS/DNA showed longer nasal residence time compared with the CS/DNA (Fig. 4A and 4B). It has been verified that prolonging antigen retention in nasal cavity is especially important for enhancing mucosal immunity [43]. Both the AL/CS/DNA and CS/DNA had more powerful bioadhesive ability compared with naked DNA, while the difference between the two NPs was not obvious enough (∼1.3 fold, Fig. 4C). The positive charged CS chains could provide strong bioadhesion with the negative charged mucosal surface like other cationic complexes [44]. Similarly, no significant difference in opening the cell tight conjunctions was observed between the two NPs (Fig. 6A). It can be explained that CS and its derivatives are considered to be able to transiently open the tight junctions between cells [45], [46], [47]. Surprisingly, enhanced intracellular uptake was verified on the AL/CS/DNA than that of the CS/DNA (Fig. 6B). Physicochemical properties such as particle size, surface charge, shape, molecular weight and composition play a key role in the cellular uptake of NPs [48]. DOPE lipid has a packing parameter value >1 and is known to adopt an inverted hexagonal structure that favors membrane destabilization for enhanced intracellular uptake. On the other hand, it is reported that globular GC (glyco chitosan) NPs showed an enhanced distribution in cells compared with the irregular GC polymers [49]. Powerful mucosal permeability and enhanced intracellular uptake results explained why more AL/CS/DNA was delivered into mucosal cells (Fig. 5B) resulting in prolonged nasal residence time, while the CS/DNA was gradually removed by mucociliary clearance. Another reason might be taken into consideration. It can be inferred that the spherical AL/CS/DNA can penetrate the mucus gels through the pores between mucin fibers to contact mucosal epithelia. However, the network shaped CS/DNA might be trapped in the luminal mucus layer because of its steric hindrance. Finally, it was gradually removed by mucociliary clearance. More details about the exact mechanism need to be explored.

It has been verified that prolonging antigen retention in nasal cavity is especially important for enhancing mucosal immunity [43]. As we expected, the AL/CS/DNA was more powerful in inducing specific mucosal and systemic responses compared with the traditional used CS/DNA system. The advantage of increased saliva anti-PAc SIgA production is obvious, which aggregates the unattached *S. mutans* and interferes with both *S. mutans* pathogenesis and colonization on the tooth surfaces. Naked DNA without delivery system via i.n. was challenged by ciliary clearance, nuclease degradation and difficulties in uptake and expression, making it unefficient in inducing mucosal and systemic immune responses. It has been indicated by several reports that, in contrast to soluble antigens, particulates could be preferentially taken up by M cells following nasal administration [50]. Noticing the relatively less populations of M cells in NALT [51] and the notorious difficulty in transfection of the APCs with non-viral vectors [52], we considered that a non M cells or APCs target strategy might be also effective in nasal mucosal immune. In fact, it has been reported that a new pathway of polymer-based DNA vaccine delivery via bystander cells without direct targeting of APCs is successful [53]. In addition, anti-PAc SIgA production in vaginal wash was evaluated (Fig. S4). The result confirmed that the AL/CS/DNA can induce a distal mucosal immune response which is agreed with most studies, because the stimulated lymphocytes in NALT can distribute to other mucosal sites such as vagina. This result also confirmed the advantage of incorporating AL.

Finally, the AL/CS/DNA showed minimal cytotoxicity and favored quick clearance via digestive tract (Fig. S5). No obvious distribution of the AL/CS/DNA was detected in other organs (heart, spleen, liver and kidney) except the lung, which reminded us of paying close attention to drug adsorption in lung via i.n. administration.

## Conclusions

This study successfully developed AL/CS/DNA nanoparticle system as a potent carrier for nasal mucosal immunization. The AL/CS/DNA exhibited the capability of mucoadhesive and pH-mediated DNA release, and induced effective mucosal immune responses. In this delivery system, AL exhibited advantages in improving the intranasal delivery of traditional used CS/DNA system. Chitosan exhibited mucoadhesive property and conditioned the pH-dependent DNA release behavior. Furthermore, the AL/CS/DNA showed minimal cytotoxicity and favored clearance via digestive tract. The AL/CS/DNA NPs are a safe and effective system which might be potentially used as an adjuvant-free DNA vaccine delivery system for mucosal immune.

## Supporting Information

Figure S1 **Release profile of DNA from FITC-CS/DNA and AL/FITC-CS/DNA in PBS (pH 6.4 or 7.4) at 37°C (n = 3).**
(TIF)

Figure S2 **Schematic illustrations of the internal structures CS/DNA and AL/CS/DNA.**
(TIF)

Figure S3 **Qualitative GFP expression in the nasal mucosa of female Wistar rats after nasal administration of naked plasmid GFP, CS/GFP and AL/CS/GFP.** The bright and fluorescence images of the cryosections were obtained from rat nasal mucosa after 4 days treatment. The letter “L” denotes luminal side of the nasal mucosa.
(TIF)

Figure S4 **Anti-PAc IgA antibody responses in vaginal wash collected at week 6 after treating with different formulations and routes.** *p<0.05, **p<0.01; Mean ± SD (n = 6).
(TIF)

Figure S5 **Fluorescence distribution after intranasal administration of AL/Cy5.5-CS/DNA.** (A) Fluorescence detected in a representative mouse at different time intervals after i.n. administration. (B) Fluorescence images in various tissues including heart, lung, stomach and intestine, liver, spleen, kidney and nasal cavity after i.n. administration for 2 h. (C) Fluorescence intensity distribution in nasal cavity, lung, stomach and intestine. (D) Fluorescence detected in excrement at different time intervals.
(TIF)

Movie S1 **The three dimensional reconstruction animation of FITC-CS/DNA distribution from the apical side to depth of 17.6 µm beneath mucosa after intranasal administration for 2 h.**
(AVI)

Movie S2 **The three dimensional reconstruction animation of AL/FITC-CS/DNA distribution from the apical side to depth of 17.6 µm beneath mucosa after intranasal administration for 2 h.**
(AVI)

## References

[1] Gurunathan S, Klinman DM, Seder RA (2000) DNA vaccines: Immunology, application, and optimization. Annual Review of Immunology 18: 927–974. 10.1146/annurev.immunol.18.1.927 10837079

[2] Liu MA (1997) The immunologist’s grail: Vaccines that generate cellular immunity. PNAS 94: 10496–10498. 9380667 10.1073/pnas.94.20.10496 PMC33772

[3] Ulmer JB, Donnelly JJ, Parker SE, Rhodes GH, Felgner PL, et al (1993) Heterologous protection against influenza by injection of DNA encoding a viral protein. Science 259: 1745–1749. 8456302 10.1126/science.8456302

[4] Wang B, Ugen KE, Srikantan V, Agadjanyan MG, Dang K, et al (1993) Gene inoculation generates immune responses against human immunodeficiency virus type 1. PNAS 90: 4156–4160. 8483929 10.1073/pnas.90.9.4156 PMC46465

[5] Barackman JD, Ott G, O’Hagan DT (1999) Intranasal immunization of mice with influenza vaccine in combination with the adjuvant LT-R72 induces potent mucosal and serum immunity which is stronger than that with traditional intramuscular immunization. Infect Immun 67: 4276–4279. 10417205 10.1128/iai.67.8.4276-4279.1999 PMC96738

[6] Zhou X, Zhang X, Yu X, Zha X, Fu Q, et al (2008) The effect of conjugation to gold nanoparticles on the ability of low molecular weight chitosan to transfer DNA vaccine. Biomaterials 29: 111–117. 17905427 10.1016/j.biomaterials.2007.09.007

[7] Wang X, Zhang XY, Kang YM, Jin HL, Du XG, et al (2008) Interleukin-15 enhance DNA vaccine elicited mucosal and systemic immunity against foot and mouth disease virus. Vaccine 26: 5135–5144. 18462848 10.1016/j.vaccine.2008.03.088

[8] Khatri K, Goyal AK, Gupta PN, Mishra N, Mehta A, et al (2008) Surface modified liposomes for nasal delivery of DNA vaccine. Vaccine 26: 2225–2233. 18396362 10.1016/j.vaccine.2008.02.058

[9] Debin A, Kravtzoff R, Santiago JV, Cazales L, Sperandio S, et al (2002) Intranasal immunization with recombinant antigens associated with new cationic particles induces strong mucosal as well as systemic antibody and CTL responses. Vaccine 20: 2752–2763. 12034102 10.1016/s0264-410x(02)00191-3

[10] Guo JH, Jia R, Fan MW, Bian Z, Chen Z, et al (2004) Construction and immunogenic characterization of a fusion anti-caries DNA vaccine against PAc and glucosyltransferase I of Streptococcus mutans. J Dent Res 83: 266–270. 14981132 10.1177/154405910408300316

[11] Fan MW, Bian Z, Peng ZX, Zhong Y, Chen Z, et al (2002) A DNA vaccine encoding a cell-surface protein antigen of Streptococcus mutans protects gnotobiotic rats from caries. J Dent Res 81: 784–787. 12407095 10.1177/0810784

[12] Jia R, Guo JH, Fan MW, Bian Z, Chen Z, et al (2006) Immunogenicity of CTLA4 fusion anti-caries DNA vaccine in rabbits and monkeys. Vaccine 24: 5192–5200. 16675075 10.1016/j.vaccine.2006.03.090

[13] Xu QA, Yu F, Fan MW, Bian Z, Guo JH, et al (2005) Immunogenicity and protective efficacy of a targeted fusion DNA construct against dental caries. Caries Res 39: 422–431. 16110216 10.1159/000086851

[14] Xu QA, Yu F, Fan MW, Bian Z, Chen Z, et al (2006) Immunogenicity and persistence of a targeted anti-caries DNA vaccine. J Dent Res 85: 915–918. 16998131 10.1177/154405910608501008

[15] Wang D, Christopher ME, Nagata LP, Zabielski MA, Li H, et al (2004) Intranasal immunization with liposome-encapsulated plasmid DNA encoding influenza virus hemagglutinin elicits mucosal, cellular and humoral immune responses. J Clin Viro 31: 99–106. 10.1016/j.jcv.2004.09.013 15567101

[16] Liu GX, Xu QA, Jin J, Li YH, Jia R, et al (2009) Mucosal and systemic immunization with targeted fusion anti-caries DNA plasmid in young rats. Vaccine 27: 2940–2947. 19428904 10.1016/j.vaccine.2009.03.009

[17] Csaba N, Garcia-Fuentes M, Alonso MJ (2009) Nanoparticles for nasal vaccination. Adv Drug Deliver Rev 61: 140–157. 10.1016/j.addr.2008.09.005 19121350

[18] van der Lubben IM, Verhoef JC, Borchard G, Junginger HE (2001) Chitosan for mucosal vaccination. Adv Drug Deliver Rev 52: 139–144. 10.1016/s0169-409x(01)00197-1 11718937

[19] Khatri K, Goyal AK, Gupta PN, Mishra N, Vyas SP (2008) Plasmid DNA loaded chitosan nanoparticles for nasal mucosal immunization against hepatitis B. Int J Pharmaceut. 354: 235–241. 10.1016/j.ijpharm.2007.11.027 18182259

[20] Raghuwanshi D, Mishra V, Das D, Kaur K, Suresh MR (2012) Dendritic Cell Targeted Chitosan Nanoparticles for Nasal DNA Immunization against SARS CoV Nucleocapsid Protein. Mol Pharmaceut 9: 946–956. 10.1021/mp200553x PMC3322645 22356166

[21] Chae SY, Son S, Lee M, Jang MK, Nah JW (2005) Deoxycholic acid-conjugated chitosan oligosaccharide nanoparticles for efficient gene carrier. J Control Release 109: 330–344. 16271416 10.1016/j.jconrel.2005.09.040

[22] Mansouri S, Cuie Y, Winnik F, Shi Q, Lavigne P, et al (2006) Characterization of folate-chitosan-DNA nanoparticles for gene therapy. Biomaterials 27: 2060–2065. 16202449 10.1016/j.biomaterials.2005.09.020

[23] Douglas KL, Piccirillo CA, Tabrizian M (2006) Effects of alginate inclusion on the vector properties of chitosan-based nanoparticles. J Control Release 115: 354–361. 17045691 10.1016/j.jconrel.2006.08.021

[24] Peng SF, Yang MJ, Su CJ, Chen HL, Lee PW, et al (2009) Effects of incorporation of poly([gamma]-glutamic acid) in chitosan/DNA complex nanoparticles on cellular uptake and transfection efficiency. Biomaterials 30: 1797–1808. 19110309 10.1016/j.biomaterials.2008.12.019

[25] Lee PW, Hsu SH, Tsai JS, Chen FR, Huang PJ, et al (2010) Multifunctional core-shell polymeric nanoparticles for transdermal DNA delivery and epidermal Langerhans cells tracking. Biomaterials 31: 2425–2434. 20034662 10.1016/j.biomaterials.2009.11.100

[26] Srinivasan C, Burgess DJ (2009) Optimization and characterization of anionic lipoplexes for gene delivery. J Control Release 71: 445–462. 10.1016/j.jconrel.2009.01.022 19331848

[27] Fillion P, Desjardins A, Sayasith K, Lagace J (2001) Encapsulation of DNA in negatively charged liposomes and inhibition of bacterial gene expression with fluid liposome-encapsulated antisense oligonucleotides. BBA-Biomembranes 1515: 44–54. 11597351 10.1016/s0005-2736(01)00392-3

[28] Filion MC, Phillips NC (1997) Toxicity and immunomodulatory activity of liposomal vectors formulated with cationic lipids toward immune effector cells. BBA-Biomembranes 1329: 345–356. 9371426 10.1016/s0005-2736(97)00126-0

[29] Aramaki Y, Tomizawa H, Hara T, Yachi K, Kikuchi H, et al (1993) Stability of Liposomes *in Vitro* and Their Uptake by Rat Peyer’s Patches Following Oral Administration. Pharm Res 10: 1228–1231. 8415412 10.1023/a:1018936806278

[30] He P, Davis SS, Illum L (1998) In vitro evaluation of the mucoadhesive properties of chitosan microspheres. Int J Pharmaceut 166: 75–88.

[31] Hagenaars N, Mania M, de Jong P, Que I, Nieuwland R, et al (2010) Role of trimethylated chitosan (TMC) in nasal residence time, local distribution and toxicity of an intranasal influenza vaccine. J Control Release144: 17–24. 10.1016/j.jconrel.2010.01.027 20100528

[32] Lavertu M, Methot S, Tran-Khanh N, Buschmann MD (2006) High efficiency gene transfer using chitosan/DNA nanoparticles with specific combinations of molecular weight and degree of deacetylation. Biomaterials 27: 4815–4824. 16725196 10.1016/j.biomaterials.2006.04.029

[33] Bot A, Bona C (2002) Genetic immunization of neonates. Microbes Infect 4: 511–520. 11932202 10.1016/s1286-4579(02)01566-6

[34] Vaughan K, Rhodes GH, Gershwin LJ (2005) DNA immunization against respiratory syncytial virus (RSV) in infant rhesus monkeys. Vaccine 23: 2928–2942. 15780742 10.1016/j.vaccine.2004.10.046

[35] Liu C, Fan M, Bian Z, Chen Z, Li Y (2008) Effects of targeted fusion anti-caries DNA vaccine pGJA-P/VAX in rats with caries. Vaccine 26: 6685–6689. 18789994 10.1016/j.vaccine.2008.08.041

[36] Sandén T, Salomonsson L, Brzezinski P, Widengren J (2010) Surface-coupled proton exchange of a membrane-bound proton acceptor. PNAS 107: 4129–4134. 20160117 10.1073/pnas.0908671107 PMC2840142

[37] Lin YH, Chen CT, Liang HF, Kulkarni AR, Lee PW, et al (2007) Novel nanoparticles for oral insulin delivery via the paracellular pathway. Nanotechnology 18: 10–20.

[38] Liao ZX, Ho YC, Chen HL, Peng SF, Hsiao CW, et al (2010) Enhancement of efficiencies of the cellular uptake and gene silencing of chitosan/siRNA complexes via the inclusion of a negatively charged poly([gamma]-glutamic acid). Biomaterials 31: 8780–8788. 20800274 10.1016/j.biomaterials.2010.07.086

[39] Schaffer DV, Fidelman NA, Dan N, Lauffenburger DA (2000) Vector unpacking as a potential barrier for receptor-mediated polyplex gene delivery. Biotechnol Bioeng 67: 598–606. 10649234 10.1002/(sici)1097-0290(20000305)67:5<598::aid-bit10>3.0.co;2-g

[40] Szoka FC, Xu Y, Zelphati O (1997) How are nucleic acids released in cells from cationic lipid-nucleic acid complexes? Adv Drug Deliver Rev 24: 291–291.

[41] Akita H, Kudo A, Minoura A, Yamaguti M, Khalil IA, et al (2009) Multi-layered nanoparticles for penetrating the endosome and nuclear membrane via a step-wise membrane fusion process. Biomaterials 30: 2940–2949. 19261326 10.1016/j.biomaterials.2009.02.009

[42] England RJA, Homer JJ, Knight LC, Ell SR (1999) Nasal pH measurement: a reliable and repeatable parameter. Clin Otolaryngol 24: 67–68. 10196653 10.1046/j.1365-2273.1999.00223.x

[43] Slutter B, Bal SM, Que I, Kaijzel E, Lowik C, et al (2010) Antigen-adjuvant nanoconjugates for nasal vaccination: an improvement over the use of nanoparticles? Mol Pharmaceut 7: 2207–2215. 10.1021/mp100210g 21043518

[44] Natsume H, Iwata S, Ohtake K, Miyamoto M, Yamaguchi M, et al (1999) Screening of cationic compounds as an absorption enhancer for nasal drug delivery. Int J Pharmaceut 185: 1–12. 10.1016/s0378-5173(99)00100-3 10425360

[45] Illum L (1998) Chitosan and its use as a pharmaceutical excipient. Pharm Res 15: 1326–1331. 9755881 10.1023/a:1011929016601

[46] Hamman JH, Stander M, Kotze AF (2002) Effect of the degree of quaternisation of N-trimethyl chitosan chloride on absorption enhancement: in vivo evaluation in rat nasal epithelia. Int J Pharmaceut 232: 235–242. 10.1016/s0378-5173(01)00914-0 11790507

[47] Slutter B, Bal SM, Que I, Kaijzel E, Lowik C, et al (2010) Antigen-djuvant Nanoconjugates for Nasal Vaccination: An Improvement over the Use of Nanoparticles? Mol Pharmaceut 7: 2207–2215. 10.1021/mp100210g 21043518

[48] Chavanpatil MD, Khdair A, Panyam J (2006) Nanoparticles for cellular drug delivery: Mechanisms and factors influencing delivery. J Nanosci Nanotechno 6: 2651–2663. 10.1166/jnn.2006.443 17048473

[49] Nam HY, Kwon SM, Chung H, Lee SY, Kwon SH, et al (2009) Cellular uptake mechanism and intracellular fate of hydrophobically modified glycol chitosan nanoparticles. J Control Release 135: 259–267. 19331853 10.1016/j.jconrel.2009.01.018

[50] Illum L (2007) Nanoparticulate systems for nasal delivery of drugs: A real improvement over simple systems? J Pharm Sci 96: 473–483. 17117404 10.1002/jps.20718

[51] Jepson MA, Clark MA, Hirst BH (2004) M cell targeting by lectins: a strategy for mucosal vaccination and drug delivery. Adv Drug Deliver Rev 56: 511–525. 10.1016/j.addr.2003.10.018 14969756

[52] Nguyen DN, Green JJ, Chan JM, Longer R, Anderson DG (2009) Polymeric Materials for Gene Delivery and DNA Vaccination. Adv Mater 21: 847–867. 28413262 10.1002/adma.200801478 PMC5391878

[53] Palumbo RN, Zhong X, Wang C (2012) Polymer-mediated DNA vaccine delivery via bystander cells requires a proper balance between transfection efficiency and cytotoxicity. J Control Release 157: 86–93. 21907252 10.1016/j.jconrel.2011.08.037 PMC3260377

