# Supplementary figures and images for: Enhanced Nasal Mucosal Delivery and Immunogenicity of Anti-Caries DNA Vaccine through Incorporation of Anionic Liposomes in Chitosan/DNA Complexes
Source: PLoS One. 2013 Aug 20;8(8):e71953. doi: 10.1371/journal.pone.0071953 (PMC3748075; doi:10.1371/journal.pone.0071953)

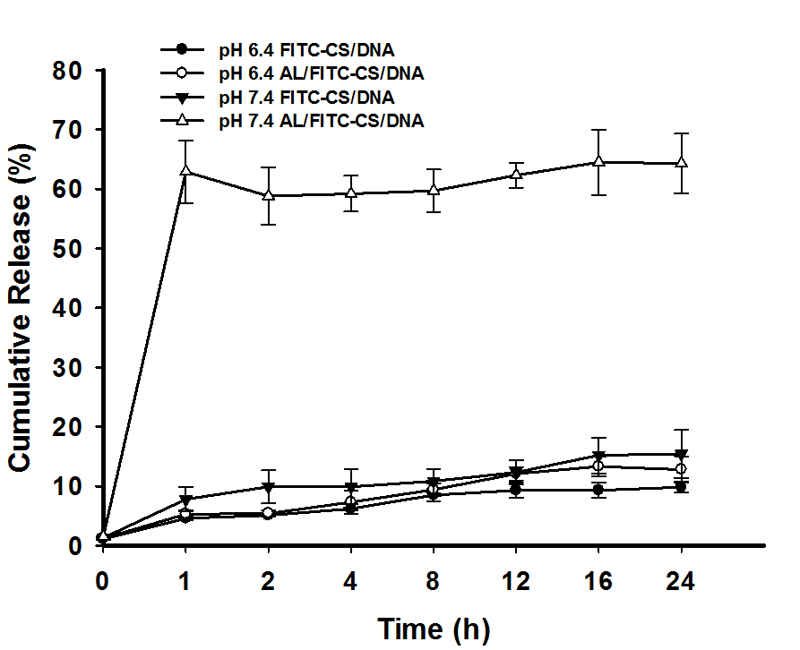

Supplement: Figure S1 — Release profile of DNA from FITC-CS/DNA and AL/FITC-CS/DNA in PBS (pH 6.4 or 7.4) at 37°C (n = 3). (TIF) [file pone.0071953.s001.tif]

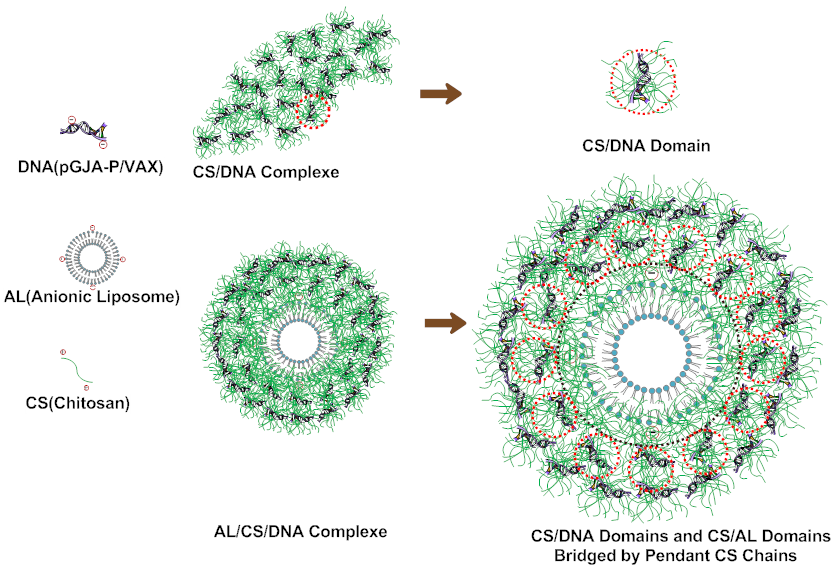

Supplement: Figure S2 — Schematic illustrations of the internal structures CS/DNA and AL/CS/DNA. (TIF) [file pone.0071953.s002.tif]

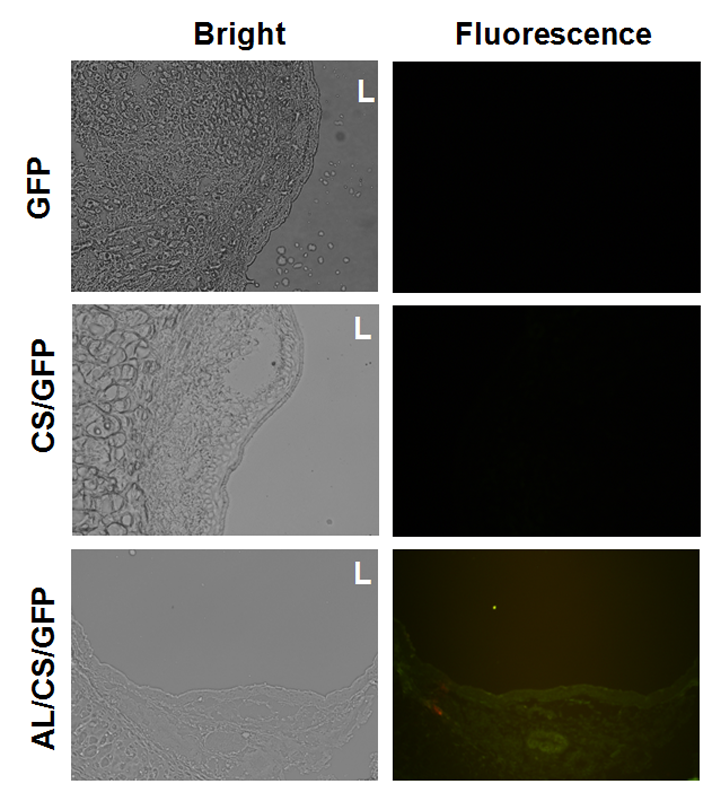

Supplement: Figure S3 — Qualitative GFP expression in the nasal mucosa of female Wistar rats after nasal administration of naked plasmid GFP, CS/GFP and AL/CS/GFP. The bright and fluorescence images of the cryosections were obtained from rat nasal mucosa after 4 days treatment. The letter “L” denotes luminal side of the nasal mucosa. (TIF) [file pone.0071953.s003.tif]

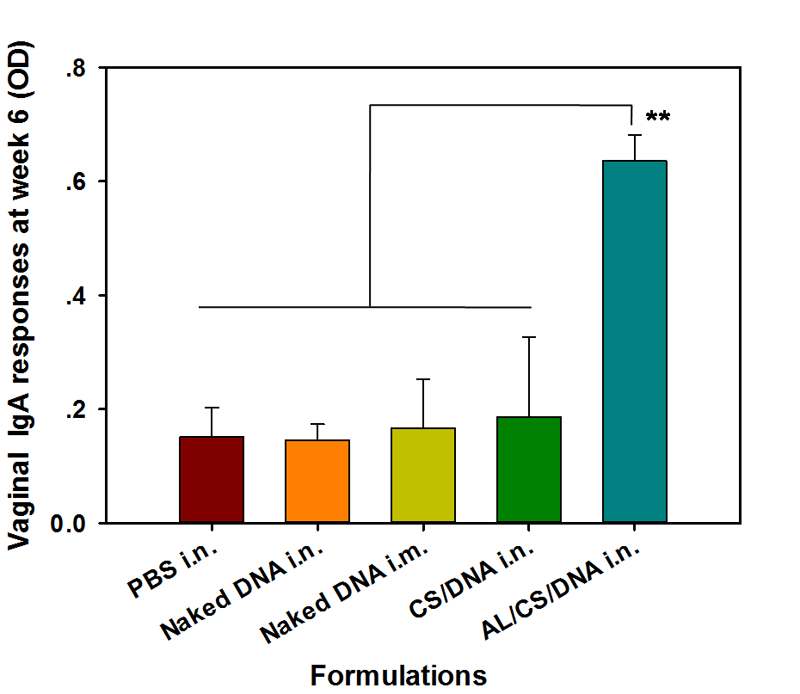

Supplement: Figure S4 — Anti-PAc IgA antibody responses in vaginal wash collected at week 6 after treating with different formulations and routes. *p<0.05, **p<0.01; Mean ± SD (n = 6). (TIF) [file pone.0071953.s004.tif]

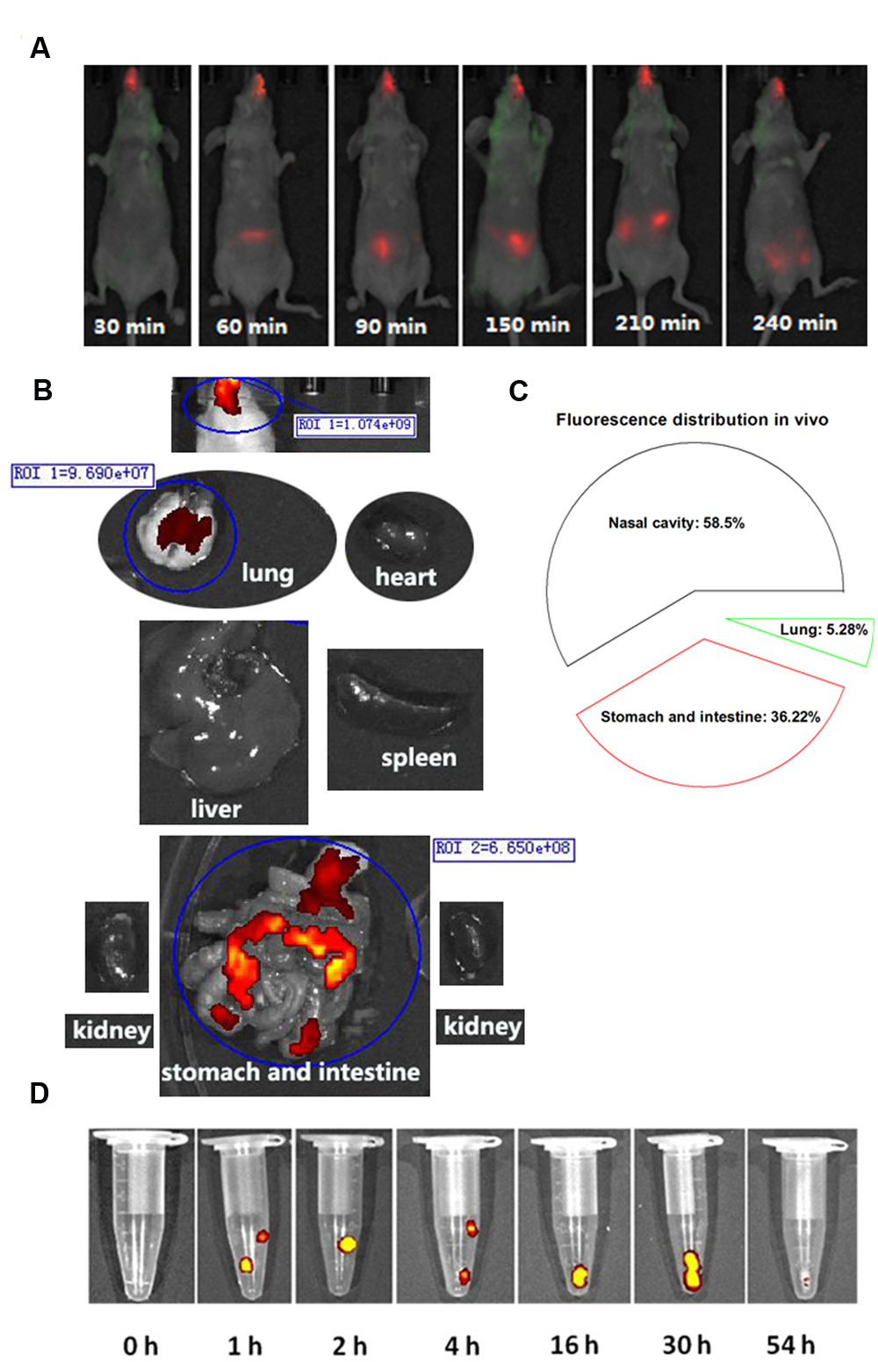

Supplement: Figure S5 — Fluorescence distribution after intranasal administration of AL/Cy5.5-CS/DNA. (A) Fluorescence detected in a representative mouse at different time intervals after i.n. administration. (B) Fluorescence images in various tissues including heart, lung, stomach and intestine, liver, spleen, kidney and nasal cavity after i.n. administration for 2 h. (C) Fluorescence intensity distribution in nasal cavity, lung, stomach and intestine. (D) Fluorescence detected in excrement at different time intervals. (TIF) [file pone.0071953.s005.tif]
